# Self-Reported Triggers Evaluation of High-Risk Dietary and Environmental Factors Preceding Migraine Onset by Using a Mobile Tracking App (Migraine Insight): Comparative Analysis Study

**DOI:** 10.2196/59951

**Published:** 2025-12-03

**Authors:** Christina Wornom, Brooklyn Brekke-Kumley, Tavsimran Luthra, Lynn J Smith, Jane C Harrington

**Affiliations:** 1 Rocky Vista University Montana College of Osteopathic Medicine Billings, MT United States; 2 Department of Medicine Albert Einstein College of Medicine Montefiore Medical Center Bronx, NY United States; 3 Migraine Insight Minneapolis, MN United States

**Keywords:** migraines, headache, environmental triggers, food triggers, food allergies, health tracking application, tracking application, application, app, environmental, migraine, food, diet, dietary, mobile tracking app, comparative analysis, user data, user, tracking

## Abstract

**Background:**

Migraines are a significant health concern affecting millions of individuals, often requiring habitual tracking of potential triggers to mitigate or predict episodes. Digital health tools such as mobile apps offer a scalable solution for personalized tracking and pattern recognition. Migraine Insight is one such app that facilitates daily logging to quantitatively assess individualized patterns of events preceding migraine onset. However, while various triggers have been reported in migraine literature, there is limited large-scale electronic user-driven data on the frequency and relative impact of specific triggers.

**Objective:**

This study aims to address this gap by analyzing user-reported data from Migraine Insight to identify the most frequently reported triggers and evaluate their potential associations with migraine onset.

**Methods:**

Food-associated migraine triggers were identified by performing a noninterventional, retrospective analysis of self-reported data obtained via the Migraine Insight app. A collaboration was made with the representatives of the Migraine Insight app to extrapolate the data needed for the study. A preliminary keyword list was assessed from a raw data set of 2605 data entry values, extracted from a 30-day period of September 19 to October 18, 2021, to identify high ranking self-reported events, classified by dietary habits, environmental conditions, body physiology, and medications. The variables examined included the following: dietary items, environmental conditions, body physiology, and electronics. Food items were combined into similar groups, considering variable spelling and descriptions of self-reported events. The association of migraine onset after consumption of top 5 dietary products was evaluated to establish the frequency of migraine episodes for all users who reported the items.

**Results:**

Collectively, food (n=353) and beverage (n=252) totaled the highest reported entries, with chocolate, wine, tea, coffee, and cheese as the highest ranked foods for prevalence of reporting across all users and for frequency of migraine onset for users who consumed the items within 48 hours. The 4 highest nonfood entries were altered sleep patterns (n=245), stress or anxiety (n=199), rain or storm conditions (n=192), and bright light or brightness (n=191). Statistical analysis showed that chocolate was the only food trigger significantly associated with migraines (*P*=.003 vs 50%; *P*=.04 vs average). Consumption of tea approached significance (*P*=.051), while consumption of coffee, cheese, and wine were not significant. These findings suggest that chocolate is the most consistent dietary trigger.

**Conclusions:**

High-risk foods, environmental conditions, stress, and lighting with highest prevalence reporting have previously been reported in medical literature, implicating that a migraine tracking app is a valid alternative to paper-based diaries.

## Introduction

Migraines are a chronic neurological condition characterized by episodic attacks that can be debilitating. Identifying potential triggers is a crucial aspect of migraine management, and health care providers often recommend symptom tracking to help patients recognize patterns. Traditionally, migraine diaries have been recorded on paper; however, retrospective reporting following an episode may introduce recall bias, leading to an overemphasis on certain factors [1]. Digital health tools such as mobile apps offer a more systematic and real-time approach to tracking potential triggers.

While migraines have been extensively studied, pinpointing specific, universal triggers remains challenging. Surveys and clinical studies suggest that common natural triggers include dietary factors, stress, weather changes, and fasting [2]. Emerging evidence highlights the role of the gut-brain axis in migraine pathophysiology, with gut microbiota influencing neurological function, stress responses, and migraine susceptibility [3,4]. A significant proportion of migraine sufferers report food-related triggers, with dairy, chocolate, and alcohol being the most frequently cited [5]. Additionally, dietary modifications such as reducing red meat and carbohydrate intake have been associated with a 30% decrease in migraine frequency and severity in certain populations [6]. Caffeine intake is another dietary factor of interest, with studies suggesting a statistically significant association between high caffeine consumption and increased migraine risk [7]. Moreover, immune-mediated responses to food allergens, including IgE-mediated reactions to eggs, milk, seafood, and animal meats, have been implicated in exacerbating migraine episodes, further supporting an inflammatory component in migraine pathogenesis [8].

Despite growing evidence linking diet to migraines, current research lacks definitive conclusions, and the complexity of dietary triggers necessitates further investigation. Additionally, unbiased and large-scale datasets are needed to assess the real-world patterns of migraine onset. Studies analyzing the effects of stress during the COVID-19 pandemic have demonstrated the value of comprehensive, real-time data collection in understanding migraine patterns [9]. Migraine Insight is a mobile app designed to help users track potential triggers, including dietary habits and lifestyle changes, independently of migraine episodes. Using machine-learning algorithms, the app generates individualized reports on high- and low-risk triggers by evaluating combinatorial factors over time.

This study aims to analyze user-reported data from the Migraine Insight app to identify the most frequently reported dietary, environmental, and behavioral triggers and assess their alignment with existing literature. We hypothesized that certain dietary items, particularly chocolate and caffeine-containing beverages, would be significantly associated with migraine episodes, and that app-based data collection would provide results consistent with previous reported triggers in the medical literature. By evaluating trigger patterns within a fixed data set, this research seeks to validate the app’s potential utility in migraine management and provide further insights into the multifactorial nature of migraine triggers.

## Methods

### Study Design

This study uses a noninterventional, retrospective analysis of self-reported data collected via the Migraine Insight mobile app (Multimedia Appendix 1). A collaboration was established with representatives from Migraine Insight to access and extract the relevant dataset. Participants voluntarily subscribed to the app, which offers both free and paid versions with varying features. All users provided informed consent in an “Opt in” manner discussed in the Apps privacy statement of self-reported deidentified data to third parties for research purposes.

An anonymized dataset containing entries from approximately 10,000 US-based users was extracted, covering a 3-year period from November 2018 to November 2021. To ensure data reliability, users who interacted with the app only once were excluded from the analysis. This exclusion was necessary because meaningful pattern recognition—especially temporal associations between triggers and migraine onset—requires multiple data points per participant. Single-use entries do not provide sufficient longitudinal information to evaluate consistent trigger-event relationships.

The dataset included user-reported migraine occurrences and associated potential triggers. Data were analyzed using Microsoft Excel. The variables examined included dietary items, environmental conditions, physiological states, and exposure to electronics. The reported food items were standardized into common categories to address variation in spelling and terminology.

Food-related triggers were classified into high-risk and low-risk groups based on the frequency with which they were reported in association with migraine events. High-risk food items were defined as those with a prevalence of ≥2% in the dataset, while low-risk items were those with <2% prevalence. The 2% threshold was chosen to balance inclusivity with relevance, highlighting commonly reported foods that could meaningfully impact population-level analyses. High-risk foods identified included coffee, tea, chocolate, wine, and cheese. Low-risk foods included meat, food additives, fruits, nuts, carbohydrates, soft beverages, beer, and high-sodium foods. The frequencies of reported high-risk foods and corresponding migraine onsets were further analyzed, with duplicates from the same user excluded to prevent skewed results from repetitive entries.

### Ethical Considerations

The investigations of this study were compliant with ethical practices established by the Nuremburg Code. Participants of the Migraine Insight tracking app provided consent for the utilization of data when agreeing to terms of use. To ensure the protection of user privacy, all identifying information was removed from the dataset prior to analysis. None of the data points, contained names, contact information, GPS coordinates, or other personally identifiable information were preserved. The anonymization process was designed to align with best practices in digital health research and data ethics, minimizing the risk of reidentification. Given the retrospective nature of the study and the use of fully anonymized, pre-existing data, the project was reviewed by the institutional review board at St. George’s University and was deemed to meet the criteria for exemption from full review. The ethical considerations in this study reflect a commitment to participant confidentiality, data security, and the responsible use of mobile health technologies in clinical research.

## Results

**Figure 1:**
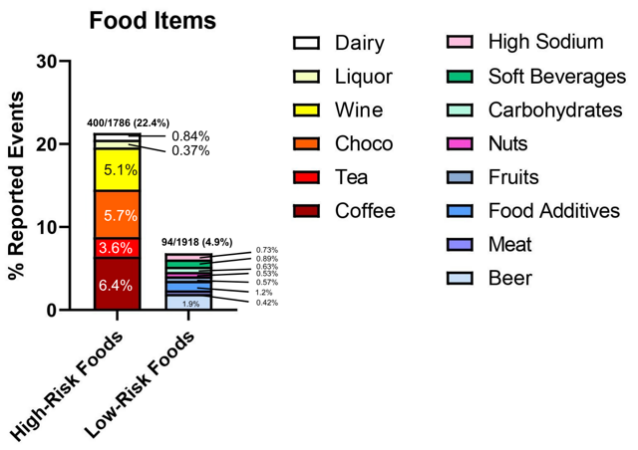
Self-reported migraine occurrence within 48 hours of consuming high-risk and low-risk food items. This stacked bar graph displays the percentage of self-reported migraines within 48 hours of consuming high-risk and low-risk food items tracked by the Migraine Insight app. Food items were combined into similar groups, considering variable spelling and descriptions of self-reported events. High-risk food items include caffeine, tea, chocolate, wine, liquor, and dairy, while low-risk food items include meat, food additives, fruits, nuts, carbohydrates, soft beverages, beer, and high-sodium–containing foods.

**Figure 2:**
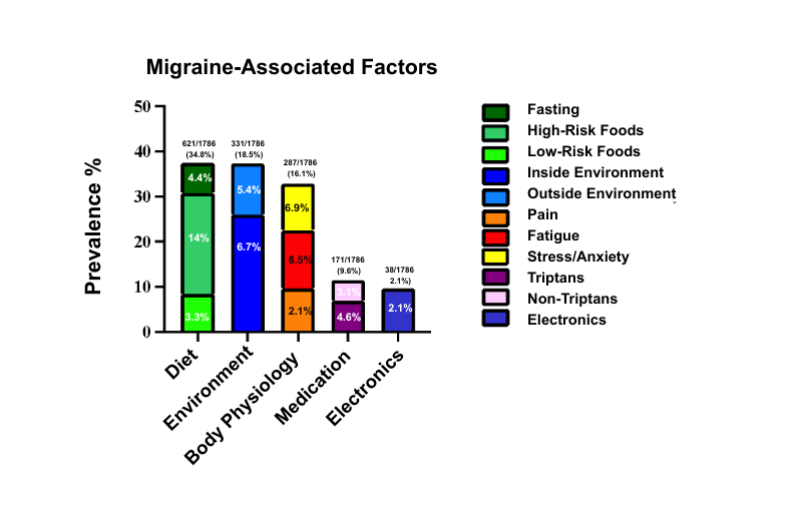
Self-reported migraine occurrence within 48 hours of encountering migraine-associated factors. This stacked bar graph depicts the percentage of self-reported migraines within 48 hours of encountering various migraine-associated factors tracked by the Migraine Insight app. The analysis grouped dietary items into high-risk food items (such as tea, coffee, chocolate, wine, liquor, and dairy), low-risk food items (such as meat, food additives, fruits, nuts, carbohydrates, soft beverages, beer and high-sodium containing foods), and fasting conditions. Environmental factors that were associated with indoor environments such as room lighting were grouped accordingly, whereas factors associated with outdoors, such as weather, were grouped under outside environment. Inside and outside environment-associated factors were grouped under environment. Physiological factors, including pain, fatigue, and stress/anxiety, were grouped into a body physiology category. Medications such as triptans and non-triptans were grouped together, while various electronic devices such as computer and phone screens were grouped under an electronics category. The grouping of dietary items was based on the consideration of variable spelling and descriptions of self-reported events.

This analysis focused on dietary triggers reported by users of the Migraine Insight app during a 30-day sampling window (September-October 2021), using a dataset derived from over 10,000 users. The app enables users to log in migraine episodes and associated lifestyle, environmental, and dietary exposures. From the raw data, entries were cleaned and grouped based on variations in spelling and terminology for consistency. Figures 1 and 2 depict the overall frequency patterns. Within 48 hours of a migraine episode, the most frequently reported categories were dietary and environmental factors. Dietary exposures were classified into high-risk food items (≥2% prevalence), low-risk food items (<2% prevalence), and fasting conditions. Reported frequencies included high-risk foods (400/1786, 22.4%), low-risk foods (94/1918, 4.9%), and fasting (127/1896, 6.7%) (Figure 1).

Among high-risk foods, caffeinated beverages (coffee and tea) were the most commonly reported, followed by chocolate, wine, and cheese. When examining the percentage of exposures that resulted in migraine onset, chocolate led with 61.8% (104/169), followed by tea (75/127, 59.4%), coffee (139/266, 52.2%), cheese (67/144, 46.8%), and wine (55/119, 46.5%). These findings are summarized in Table 1 below, which includes confidence intervals and statistical significance testing.

**Table 1:** Migraine triggers with confidence intervals and statistical significance. This table shows a summary of high-risk dietary triggers (coffee, chocolate, cheese, tea, and wine), showing exposure counts, migraine occurrence rates, 95% CIs, and *P* values. Significance was tested against a 50% threshold and the overall average (53.3%). Chocolate was the only trigger with statistically significant results in both comparisons (*P*<.05).

Trigger	Total exposures, n	Resulted in migraine, n	No migraine, n	Resulted in migraine (%) (95% CI)	*P* (vs 50%)	Sig vs 50%^a^	*P* value vs average	Sig vs avg^b^
Coffee	266	139	127	52.2 (46.3-58.2)	.50	^c^	.76	^c^
Chocolate	169	104	65	61.8 (54.0-68.5)	.003	^d^	.04	^d^
Cheese	144	67	77	46.8 (38.6-54.7)	.45	^c^	.11	^c^
Tea	127	75	52	59.4 (50.4-67.2)	.05	^c^	.21	^c^
Wine	119	55	64	46.5 (37.5-55.2)	.46	^c^	.14	^c^
Overall	825	440	385	46.5 (37.5-55.2)	.06	^c^	N/A^e^	N/A

^a^Tests if trigger differs from chance (50%) versus average.

^b^Tests if trigger differs from overall average across all triggers.

^c^Statistically not significant versus 50%.

^d^Statistically significant at *P*<.05.

^e^N/A: not applicable.

Statistical analysis using proportion tests (binomial test vs 50% and vs overall average of 53.3%) revealed that chocolate was the only food trigger that was statistically significant compared to both the 50% threshold (*P*=.003) and the overall average (*P*=.04). Tea approached significance compared to 50% (*P*=.05) but did not meet the conventional *P*<.05 threshold. No other individual food reached statistical significance, including coffee, cheese, and wine. These findings suggest that while all 5 foods were commonly reported prior to migraines, chocolate stands out as a more consistently associated trigger, both by user reporting (Table 2) and statistical analysis (Table 1).

**Table 2:** Frequency of migraine occurrence within 48 hours of consuming certain food items.

Food item	Users tracking the trigger, n	Average trigger resulting in a migraine within 48 h, n (%)
Chocolate	169	104 (61.8)
Tea	127	75 (59.4)
Coffee	266	139 (52.2)
Cheese	144	67 (46.8)
Wine	119	55 (46.5)

Table 2 displays the average percentage of times users of the Migraine Insight app reported experiencing a migraine within 48 hours of consuming specific food items, along with the number of users that are tracking that food item. The data were collected through self-reported triggers and tracked by the app. The food items listed were reported as common triggers by many users.

Nondietary triggers were also assessed. Among these, altered sleep patterns (n=245), stress or anxiety (n=199), rain/storm conditions (n=192), and bright light (n=191) were the most frequently reported within 48 hours of migraine onset. These environmental and behavioral factors were frequently reported independently or in conjunction with dietary triggers, though formal multivariate analysis was not conducted in this study.

Physiological triggers, including fatigue (n=245) and pain (n=61), were grouped into a broader “body physiology” category. Medication use, including both triptans (n=131) and non-triptan medications (n=88), and screen exposure (eg, phone, computer use) were also reported. Triptans were captured through various spellings and brand names (eg, Sumatriptan, Rizatriptan), and non-triptan medications included over the counter and prescription agents such as hydroxyzine, Zomig, Toradol, and prochlorperazine.

## Discussion

### Principal Findings

This study identifies high-risk environmental, dietary, and behavioral factors preceding migraine onset by using data from the Migraine Insight app. The patterns observed align with previous research highlighting certain foods and environmental conditions as potential migraine triggers. High-risk items, including chocolate, tea, coffee, wine, and cheese, emerged as significant contributors, corroborating findings by Martin and Vij [10,11], who identified chocolate, caffeine, and alcohol as common dietary triggers. Similarly, data from Migraine Insight users support research by Nowaczewska et al [12], emphasizing the role of chocolate and aged cheese in migraine risk.

Among dietary triggers, tea and coffee were the second and third most frequently reported triggers (Table 2). There were 127 reports of tea triggering migraines from 45 users, 9 of whom also reported coffee as a trigger. In total, 123 users reported migraines triggered by coffee, with 266 occurrences. Tea consumption led to migraines in 59.4% (75/127) of the users who reported its intake, while coffee triggered migraines in 52.2% (139/266) of the users. One possible explanation for tea’s higher likelihood of inducing migraines is its greater tannin content compared to coffee. Tannins, a type of flavonoid phenol, are found in various dietary sources [13]. A study analyzing tannin levels in both beverages found that roasted coffee beans contain 18 (SD 1.7) mg·g⁻¹ of tannins, whereas tea contains nearly twice that amount at 37 (SD 2.6) mg·g⁻¹ [14]. Tannins are also present in high concentrations in chocolate, wine, and dairy—all of which were frequently reported as migraine triggers [15].

Beyond dietary influences, nondietary factors such as weather, light, and stress have been widely recognized as migraine triggers. Stress, in particular, is commonly cited, with a study of over 1200 participants reporting that 80% identified stress as a migraine trigger [16-18]. Similarly, an analysis of 1027 individuals who met the International Headache Society's migraine criteria found that 53.2% reported weather changes as a precipitating factor [19]. Another study identified weather as the second most frequently perceived trigger after stress [18]. Light exposure also emerged as a significant factor, particularly among migraineurs with aura, where 75% identified light as a trigger compared to 35% of those without aura [20].

The use of a mobile tracking app for data collection represents a novel and valuable approach in migraine research. Unlike traditional paper-based diaries, which are prone to recall bias, real-time data logging offers a more accurate and comprehensive view of potential triggers. This method, as demonstrated in our study, aligns with existing literature and enhances the reliability of self-reported data [7,21]. Furthermore, the impact of COVID-19 lockdowns on stress-induced migraine attacks, as studied by Kato et al [9], underscores the value of leveraging unbiased datasets in migraine research.

While this study provides meaningful insights into the dietary, environmental, and behavioral factors preceding migraines, certain limitations must be acknowledged. The reliance on self-reported data introduces the potential for recall bias, as participants may not accurately remember or report their exposures. Additionally, the self-selecting nature of the participant pool may lead to selection bias, limiting the generalizability of the findings. Although mobile apps provide real-time data collection that reduces retrospective inaccuracies, they also introduce challenges such as inconsistent user engagement and variations in reporting accuracy. Traditional methods such as structured clinical assessments or controlled dietary studies may offer more standardized data but lack the real-world applicability and convenience of mobile tracking. Future research should aim to balance these methodologies by incorporating objective biomarkers or clinician-verified reports alongside mobile app data. Expanding the participant base to encompass more diverse populations and employing more controlled methodologies will further strengthen the validity of these findings. Addressing these gaps will contribute to a deeper understanding of migraine pathophysiology and improved management strategies.

### Conclusion

This study highlights the potential of mobile health apps such as Migraine Insight to identify high-risk dietary, environmental, and behavioral factors associated with migraine onset. The findings validate existing literature by confirming that commonly cited triggers—especially chocolate, tea, and coffee—remain prominent among real-world users. Notably, chocolate demonstrated a statistically significant association with migraine episodes, reinforcing its relevance in dietary counseling for migraine prevention.

Beyond individual triggers, this research underscores the utility of app-based, real-time data collection as a reliable alternative to traditional migraine diaries. The ability to capture consistent, personalized data offers new opportunities for understanding complex, multifactorial patterns in migraine occurrence. While limitations related to self-reporting, recall bias, and user engagement remain, the insights gained from large-scale, user-generated datasets provide a strong foundation for further investigation.

Future research should aim to integrate app-based tools with clinical assessments and objective measures such as biomarkers to enhance validity. Expanding participation to include more diverse populations and exploring longitudinal outcomes will further strengthen the evidence base. Ultimately, mobile tracking technologies hold significant promise in advancing personalized migraine management and improving the quality of life for individuals living with these conditions.

## Data Availability

The datasets generated or analyzed during this study are available from the corresponding author on reasonable request.

## Appendix

Multimedia Appendix 1 Analysis of the triggers.

Multimedia Appendix 2 Document containing original prompts/responses on file from ChatGPT used in the editing of this paper.
