# Supplementary material for: Self-Reported Triggers Evaluation of High-Risk Dietary and Environmental Factors Preceding Migraine Onset by Using a Mobile Tracking App (Migraine Insight): Comparative Analysis Study
Source: JMIR Form Res. 2025 Dec 3;9:e59951. doi: 10.2196/59951 (PMC12712558; doi:10.2196/59951)
Supplement: Multimedia Appendix 2 [file formative_v9i1e59951_app2.docx]

can you help me with editing a research manuscript? I'm going to give you the abstract and the comments i got from the reviewer

**ChatGPT said:**

Of course! Send me the abstract and the reviewer comments, and I’ll help you refine your manuscript accordingly.

**You said:**

Introduction: Migraines are a significant health concern for millions of patients who are reliant on habitual tracking of event triggers, with the goal of reducing or predicting episodes. Migraine Insight is an app that facilitates daily logging to quantitatively determine individualized patterns of events preceding migraine onset. To investigate patterns for most frequently reported triggers from user data of the Migraine Insight app user entries were evaluated.

**ChatGPT said:**

Got it! Now send over the reviewer comments, and I’ll help you revise the introduction accordingly.

**You said:**

here is the feedback. Abstract: The abstract is comprehensive but lacks details about the statistical methods used. Including a brief mention of the specific tests or analyses performed would improve clarity. Introduction: While the introduction provides a good overview of the condition, it could benefit from a clearer research gap statement. What specific aspect of migraine triggers is understudied or novel in this study?

**ChatGPT said:**

Here’s a revised version of your introduction incorporating the reviewer’s feedback:

**Introduction:** Migraines are a significant health concern affecting millions of individuals, often requiring habitual tracking of potential triggers to mitigate or predict episodes. Digital health tools, such as mobile applications, offer a scalable solution for personalized tracking and pattern recognition. *Migraine Insight* is one such app that facilitates daily logging to quantitatively assess individualized patterns of events preceding migraine onset. However, while various triggers have been reported in migraine literature, there is limited large-scale user-driven data on the frequency and relative impact of specific triggers. This study aims to address this gap by analyzing user-reported data from *Migraine Insight* to identify the most frequently reported triggers and evaluate their potential associations with migraine onset.

This revision clarifies the research gap and strengthens the rationale for your study. Let me know if you’d like further refinements! Also, if you provide your methods section, I can help incorporate the missing statistical details in the abstract.

**You said:**

that is great. next, can you help me revise my introduction based on feedback from the reviewer?

**ChatGPT said:**

Sure! Send me the full introduction along with any specific reviewer comments, and I’ll help refine it accordingly.

**You said:**

INTRODUCTION Migraines are a chronic condition with debilitating effects to those who experience sporadic episodes, preceded by a variety of triggers. Neurologists and primary care physicians encourage people to track migraine symptoms, medication usage, and triggers in between appointments. Standard practice stipulates that diaries are recorded on paper; however, recording of retrospective events after a migraine episode can potentially lead to biased emphasis on factors [1]. While the condition has been well defined with objective parameters, studies unilaterally pointing to specific causes are relatively unknown. Surveys and trials show there are several possible natural triggers of migraines including diet, stress, weather change, and fasting [2]. Previous research has established a bidirectional interaction between the central nervous system and the enteric nervous system called the gut/brain axis. Additionally, the gut microbiota is found to play a role in brain function and can affect emotional behavior, stress management and migraine attacks [3,4]. A significant proportion of migraineurs report that migraine attacks can be triggered by certain dietary items, most frequently dairy, chocolate, and alcohol [5]. In a study of 204 migraineurs, reduced consumption of red meats and carbohydrates showed a 30% decrease in migraine frequency and severity [6]. Caffeine consumption is also an area to be investigated. In a study of 101 migraineurs, a statistically significant association suggested that high caffeine intake could trigger migraines [7]. Furthermore, an IgE-mediate immunological response to food allergens, including egg, milk, seafood, and animal meats, exacerbate frequency and intensity of migraine episodes, suggesting an inflammatory cytokine contribution to migraine pathophysiology [8]. The relationship between diet and migraines is complex and multifactorial. As evidenced by our findings and supported by Altamura et al., dietary modification, such as reducing consumption of red meats and carbohydrates, can significantly decrease migraine frequency and severity [6]. This suggests a potential bidirectional interaction between the central nervous system and the enteric nervous system, commonly referred to as the gut-brain axis [4]. The influence of gut microbiota on brain function and migraine attacks further highlights the intricate connection between dietary intake and migraine pathophysiology [3]. Although current studies make no definite conclusions on food triggers, evidence suggests that diet can be a tool for controlling migraine attacks and that further studies in this area would be very beneficial. An examination of the impacts of COVID-19 pandemic lockdowns on stress-induced migraine attacks demonstrated the benefit of unbiased data sets to assess patterns of migraine onset [9]. Migraine Insights is a mobile-based application that allows users to report events, such as altered sleep, or dietary habits, such as consumption of coffee, in habitual daily manner, independent of migraine episodes and creates an individualized report of high and low risk triggers with algorithmic machine learning that considers combinatorial factors. The aims of this study were to evaluate trigger patterns across a data set extracted for a fixed period and to compare the high risk reported events to established literature on migraine as a validity of the application.

**ChatGPT said:**

Here’s a revised version of your introduction that addresses the reviewer’s feedback. I improved the clarity of the research gap, removed redundancy, and enhanced the logical flow while maintaining the original intent of your study.

**Introduction**
Migraines are a chronic neurological condition characterized by episodic attacks that can be debilitating. Identifying potential triggers is a crucial aspect of migraine management, and healthcare providers often recommend symptom tracking to help patients recognize patterns. Traditionally, migraine diaries have been recorded on paper; however, retrospective reporting following an episode may introduce recall bias, leading to an overemphasis on certain factors [1]. Digital health tools, such as mobile applications, offer a more systematic and real-time approach to tracking potential triggers.

While migraines have been extensively studied, pinpointing specific, universal triggers remains challenging. Surveys and clinical studies suggest that common natural triggers include dietary factors, stress, weather changes, and fasting [2]. Emerging evidence highlights the role of the gut-brain axis in migraine pathophysiology, with gut microbiota influencing neurological function, stress responses, and migraine susceptibility [3,4]. A significant proportion of migraine sufferers report food-related triggers, with dairy, chocolate, and alcohol being the most frequently cited [5]. Additionally, dietary modifications—such as reducing red meat and carbohydrate intake—have been associated with a 30% decrease in migraine frequency and severity in certain populations [6]. Caffeine intake is another dietary factor of interest, with studies suggesting a statistically significant association between high caffeine consumption and increased migraine risk [7]. Moreover, immune-mediated responses to food allergens, including IgE-mediated reactions to eggs, milk, seafood, and animal meats, have been implicated in exacerbating migraine episodes, further supporting an inflammatory component in migraine pathogenesis [8].

Despite growing evidence linking diet to migraines, current research lacks definitive conclusions, and the complexity of dietary triggers necessitates further investigation. Additionally, unbiased and large-scale datasets are needed to assess the real-world patterns of migraine onset. Studies analyzing the effects of stress during the COVID-19 pandemic have demonstrated the value of comprehensive, real-time data collection in understanding migraine patterns [9]. *Migraine Insight* is a mobile application designed to help users track potential triggers, including dietary habits and lifestyle changes, independently of migraine episodes. Using machine-learning algorithms, the app generates individualized reports on high- and low-risk triggers by evaluating combinatorial factors over time.

This study aims to analyze user-reported data from *Migraine Insight* to identify the most frequently reported triggers and assess their alignment with existing literature. By evaluating trigger patterns within a fixed data set, this research seeks to validate the application’s potential utility in migraine management and provide further insights into the multifactorial nature of migraine triggers.

**Key Improvements:**

✅ **Clearer Research Gap:** Emphasized the lack of definitive conclusions on migraine triggers and the need for large-scale, unbiased data analysis.
✅ **Logical Flow:** Restructured the introduction for better coherence and removed redundant statements.
✅ **Stronger Justification:** Highlighted the importance of digital tracking tools in migraine research.

Would you like me to refine any specific parts further?
